# *Erratum*: Vol. 63, No. SS-11

**DOI:** 10.15585/mmwr.mm6948a7

**Published:** 2020-12-04

In the Surveillance Summary “Abortion Surveillance — United States, 2011,” data on the number of known previous induced abortions for women having abortions in this reporting year were erroneously included for New York City. These data did not meet reporting standards and should have been excluded from this report. When corrected, among women with abortions in the reporting year, the proportion with no previous induced abortions increased, and the proportion with one or more previous induced abortions decreased.

On page 8, the first paragraph should have read “Data from the **37** areas that reported the number of previous abortions for women who obtained abortions in 2011 indicate that the majority (**56.9%**) had no previous abortions, **36.1%** had one to two previous abortions, and **7.1%** had three or more previous abortions (Table 19). Among the **30** reporting areas^¶¶¶¶^ that provided data for the relevant years of comparison (2002 versus 2006, 2007 versus 2011, and 2010 versus 2011), the percentage of women who had one to two previous abortions was stable, although there was a decrease among women who had zero previous abortions and an increase among women who had three or more previous abortions. Among the areas included in this comparison, **57.8%**, **36.0%**, and **6.2%** of women had zero, one to two, or three or more previous abortions, respectively, in 2002; by contrast, **57.0%**, **35.9%**, and **7.2%** of women had zero, one to two, or three or more previous abortions, respectively, in 2011.” New York City should have been included in the ^¶¶¶¶^ footnote, which lists reporting areas that were not included in these estimates.

In Table 19, the line for New York City should be deleted. For the total line, the numbers and percentages should have read **229,909 (56.9), 102,612 (25.4), 43,159 (10.7), 28,593 (7.1), 404,273 (97.8)**. The * footnote should have read “Data from **37** reporting areas; excludes **15** areas (California, Connecticut, District of Columbia, Florida, Georgia, Illinois, Maryland, New Hampshire, New Mexico, **New York City**, New York State, North Carolina, Vermont, Wisconsin, and Wyoming) that did not report, did not report by the number of previous induced abortions, or did not meet reporting standards.” The total in the ** footnote should have been **413,504**.

